# Using phosphine ligands with a biological role to modulate reactivity in novel platinum complexes

**DOI:** 10.1098/rsos.171340

**Published:** 2018-02-21

**Authors:** Marcelo Echeverri, Amparo Alvarez-Valdés, Francisco Navas, Josefina Perles, Isabel Sánchez-Pérez, A. G. Quiroga

**Affiliations:** 1 Inorganic Chemistry Department, Universidad Autónoma de Madrid, Madrid, Spain; 2 Single Crystal XRD Laboratory, SIdI, Universidad Autónoma de Madrid, Madrid, Spain; 3 Instituto de Investigaciones Biomédicas Albert Sols, Madrid, Spain

**Keywords:** metallodrug–protein interaction, cytotoxicity, platinum drugs and DNA

## Abstract

Three platinum complexes with *cis* and *trans* configuration *cis*-[Pt(TCEP)_2_Cl_2_], *cis*-[Pt(tmTCEP)_2_Cl_2_] and *trans*-[Pt(TCEP)_2_Cl_2_], where TCEP is tris(2-carboxyethyl)phosphine, have been synthesized and fully characterized by usual techniques including single-crystal X-ray diffraction for *trans*-[Pt(TCEP)_2_Cl_2_] and *cis*-[Pt(tmTCEP)_2_Cl_2_]. Here, we also report on an esterification process of TCEP, which takes place in the presence of alcohols, leading to a platinum complex coordinated to an ester tmTCEP (2-methoxycarbonylethyl phosphine) ligand. The stability in solution of the three compounds and their interaction with biological models such as DNA (pBR322 and calf thymus DNA) and proteins (lysozyme and RNase) have also been studied.

## 1. Introduction

The discovery of new metallodrugs that can overcome the unwanted effects produced by cisplatin [1] (currently the better known metallodrug in the clinic) or carboplatin has led to novel platinum complexes with non-conventional design. Some of those non-conventional examples go against the *cis* configuration requirements established by cisplatin [2]; for example, new active species are possible using [*trans-*PtX_2_(L)(L′)] complexes. Metallodrugs such as organometallic AuIII/RuII/OsII/RhIII/IrIII complexes have emerged as a new trend in non-conventional metallodrugs due to their potential activation by the redox balance in cancer cells and/or their protein interaction [3,4].

*Trans* complexes have been studied in detail, revealing that synthetic variations of transplatin in the coordinating ligands X and L [2,5] conferred cytotoxic activity in particular for cisplatin-resistant cell lines. Several structural motifs have been explored within these variations including diamine complexes [6], polyplatinum compounds, photoactivatable azide complexes [7], intercalator-linked species [8,9] and monofunctional compounds [10]. Among these examples, we are especially interested in aliphatic amine compounds with phosphines in *trans* configuration.

The use of these bulky ligands with hydrophobic properties can produce steric impediments and may be the cause of the increased cytotoxicity. Phosphines are then a good group to design not only catalysts but also potential metallodrugs. Looking for phosphines with biological properties that could improve the pharmacokinetics of a platinum complex, we found that tris(2-carboxyethyl)phosphine (TCEP) is a soluble phosphine, widely used in biochemical systems [11]. Its esterification derivative, tris(2-methoxycarbonylethyl)phosphine (tmTCEP), showed even higher permeability than TCEP through the cell membrane [11] and even higher reactivity as a reducing agent. An interesting biological interaction has been reported for TCEP in the cisplatin–protein adduct formation with Atox1 [12], a chaperon protein related to copper transport inside the cell. The isolation and X-ray characterization of an adduct with cisplatin and this protein helped in the understanding of this drug reactivity inside the cell [13].

The use of TCEP as a phosphine-coordinating ligand could improve not only the solubility of platinum compounds, but also their transport through the cell and even their secondary effects. We have synthesized, characterized and compared with previous results [14] *cis* and *trans* complexes using TCEP and tmTCEP.

## 2. Results and discussion

### 2.1. Chemistry

**Scheme 1.**
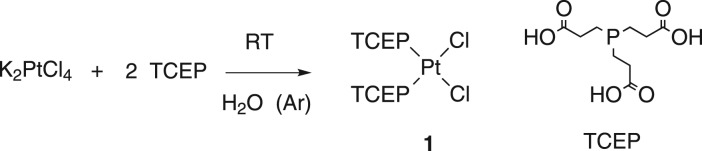
Formation reaction of *cis*-[Pt(TCEP)_2_Cl_2_.

The reaction of tetrachloroplatinate with TCEP in water leads to the formation of the *cis*-[Pt(TCEP)_2_Cl_2_] (**1**) complex (scheme 1). The absence of oxygen is a very important condition for the isolation of the pure compound; otherwise, the TCEP oxidizes. Monitoring the reaction by ^31^P-NMR detects a new signal at 15.9 ppm highly shifted from the free ligand signal at 56.7 ppm.

The characterization of complex **1** was performed by the usual techniques and the detailed data are compiled in the Experimental part. All the techniques indicate that the structural proposal adjusts to the general formula *cis*-[Pt(TCEP)_2_Cl_2_]; only one signal in the ^31^P-NMR and ^195^Pt-NMR spectra at 4.87 and at −4420.5 ppm, respectively [15], confirmed the PtCl_2_P_2_ coordination and the two *ν*(Pt–Cl) stretching bands in the IR spectrum at 261 and 361 cm^−1^ confirm the *cis* configuration.

Our next step was the esterification of TCEP to get tmTCEP and the corresponding complex. We found the esterification reported in the literature, and it was carried out in MeOH in the presence of a cation-exchange resin followed by a preparative HPLC purification [11].

**Scheme 2.**
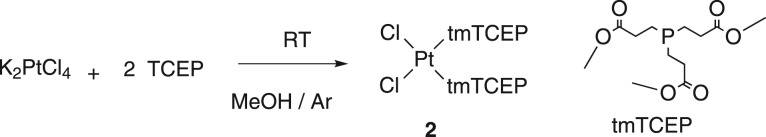
Formation reaction of *cis*-[Pt(tmTCEP)_2_Cl_2_.

In our method, we have avoided these tedious stages and proceed with both esterification and platinum complex formation in only one step. The reaction of K_2_PtCl_4_ with two equivalents of TCEP using MeOH as a solvent leads to a quantitative formation of *cis*-Pt(tmTCEP)_2_Cl_2_ (**2**; scheme 2).

**Figure 1.**
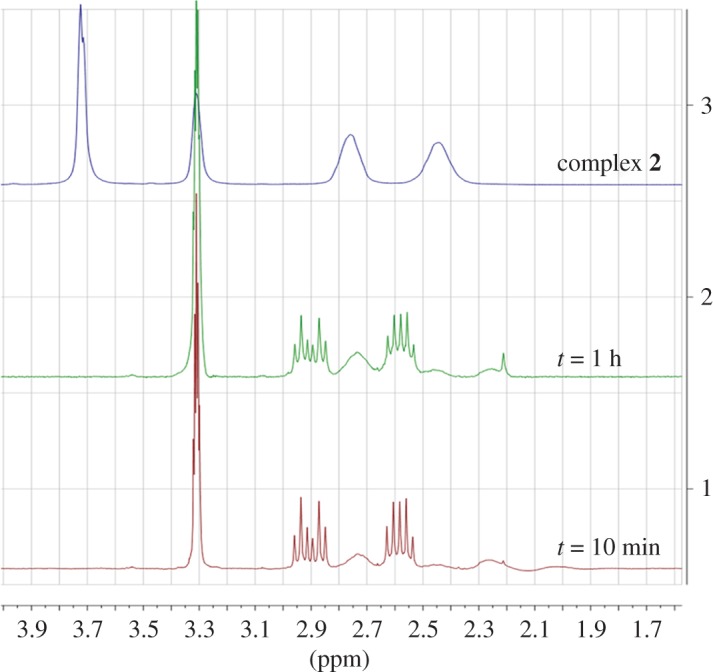
Progress of the reaction of scheme 2 as monitored by ^1^H-NMR spectroscopy with regard to the time: 10 min, 1 h in MeOH solution and pure complex **2** (at the top).

We monitored the reaction by ^1^H-NMR (figure 1) and observed the beginning of the reaction already after 10 min, where a new set of signals arises corresponding to the tmTCEP coordinated to the platinum atom. The final complex **2** is the major species after 24 h. Following the same procedure as with the previous complexes, we used the usual techniques to characterize complex **2**, with all details available in the Experimental part and the electronic supplementary material.

The characterization by NMR showed clearly the esterification, with a new signal corresponding to the methoxy group at 3.72 ppm in the ^1^H-NMR spectrum and at 52 ppm in the ^13^C-NMR spectrum.^195^Pt-NMR, at −4433 ppm, is in agreement with a Pt atom bound to phosphorus and chloride atoms [15]. The lack of the *ν*(O–H) stretching band and the *ν*(C=O) at 1734 cm^−1^ in the complex **2** IR spectrum support the esterification of the TCEP ligand. The two *ν*(Pt–Cl) bands at 283 and 317 cm^−1^ are in accordance with a *cis* configuration (C_2 V_ symmetry).

**Scheme 3.**
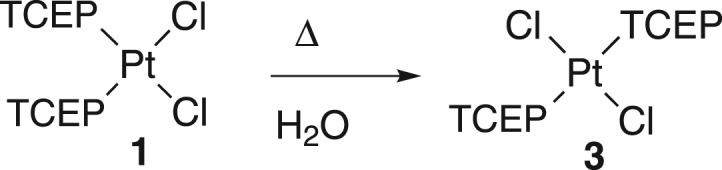
Isomerization of complex **1** to complex **3**.

The stability of the complex solutions in water was analysed for complexes **1**, **2** and **3**. The *cis* complex **1** in water solution slowly isomerizes to the *trans* counterpart **3** (scheme 3). This process takes place very slowly and it can be accelerated starting with a very concentrated solution and increasing the temperature (50°C).

We detected no changes for complex **2** and **3** solutions (dimethylsulfoxide (DMSO) and D_2_O solutions) when monitoring the solution by ^1^H-NMR over 24 h. UV--visible spectrophotometric analysis allowed the calculation of the constants (table 3) based on the absorption profile plottings as a function of time (included in electronic supplementary material, figure S1) [16]. The stability of the *cis* complex is higher than that of the *trans* counterpart. This trend is consistent with a more rapid aquation step for the *trans* configuration [17]. (*K* (s^−1^) values have been compiled with the protein interaction in table 3.)

Complex **3** was also fully characterized by the usual techniques which allowed one to confirm the *trans*-[Pt(TCEP)_2_Cl_2_] configuration with only one active *ν*(Pt–Cl) stretching band in the IR spectrum at 325 cm^−1^ expected for a D_2 h_ symmetry group. ^31^P-NMR and ^195^Pt-NMR signals at 9.25 and at −4357.5 ppm [15] showed a clear difference with the *trans* counterpart.

### 2.2. Structural study

Compounds **2** and **3** were obtained as crystals suitable for single-crystal X-ray diffraction. Unfortunately, the many attempts performed for the recrystallization of complex **1** did not afford good results, and we were not able to obtain the X-ray structure of this *cis* complex, although this compound had previously been obtained as a solvate ([*cis*-PtCl_2_(TCEP)_2_]·1,25H_2_O) and its structure had been solved [14]. Compound **1** might be difficult to obtain as single crystals because of its slow isomerization in water solution to the *trans* counterpart, complex **3**.

**Figure 2.**
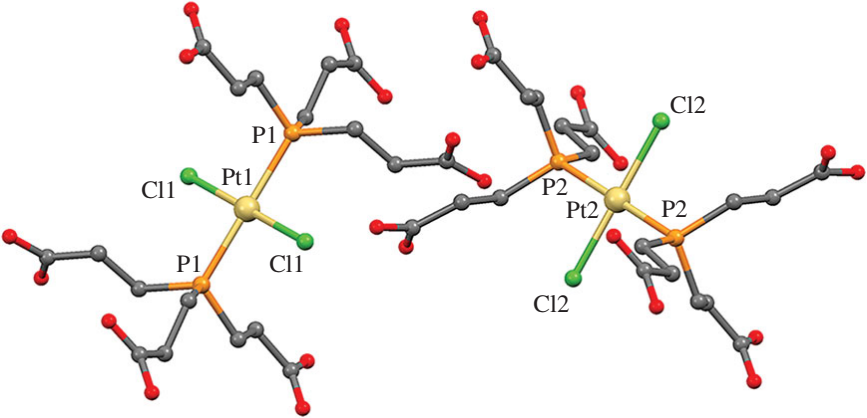
Molecular view of the two crystallographically independent molecules found in the structure of complex **3**. Hydrogen atoms have been omitted for clarity.

We have carefully compared the crystal structure of complex **3** (figure 2) with the deuterated form (*trans*-[PtCl_2_{P(C_2_H_4_COOD)_3_}_2_]) described in the literature [14], and found that both are isostructural. Compound **3** crystallizes in the triclinic *P*-1 space group with two halves of *trans*-Pt(tmTCEP)_2_Cl_2_ molecules per asymmetric unit. Both Pt1 and Pt2 platinum atoms show a square planar geometry with the expected angles for this coordination environment. Distances Pt–Cl are slightly longer than those found in the literature for similar *trans* compounds, and in the range 2.301–2.317 Å [18,19]; Pt–P distances are also within the expected range of 2.315–2.373 Å [18,20] (table 1). The supramolecular packing in the crystal is mainly achieved by double O⋯HO interactions (see electronic supplementary material, table 1) of the six terminal carboxylic groups with those of neighbouring molecules, giving rise to a very interesting supramolecular network.

**Table 1.** Selected distances and angles for complex **3**. Symmetry operations: #1 − *x*, −*y* + 1, −*z* + 2; #2 −*x* + 1, −*y* + 1, −*z* + 1.

bond distances (Å)		bond angles (°)			
Pt1–P1	2.311(1)	P1–Pt1–P1#2	180	P1–Pt1–Cl1	93.47(5)
Pt1–Cl1	2.337(1)	P1–Pt1–Cl1#2	86.53(5)	P1#2–Pt1–Cl1#2	93.47(5)
		P1#2–Pt1–Cl1	86.53(5)	Cl1–Pt1–Cl1#2	180
Pt2–P2	2.296(1)	P2–Pt2–P2#1	180	P2#1–Pt2–Cl2#1	86.57(5)
Pt2–Cl2	2.336(1)	P2–Pt2–Cl2#1	93.42(5)	P2#1–Pt2–Cl2	93.43(5)
		P2–Pt2–Cl2	86.58(5)	Cl2#1–Pt2–Cl2	180

**Figure 3.**
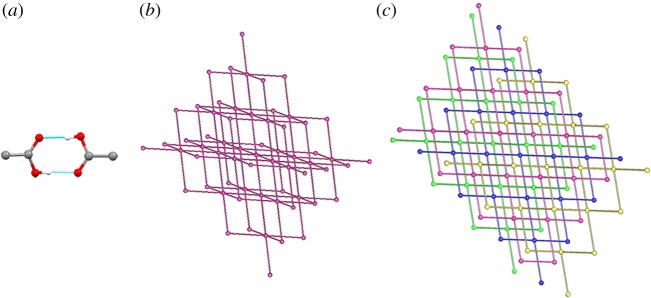
(*a*) Details of the double hydrogen bond between neighbour carboxylic acid groups found in **3**. (*b*) Single underlying **pcu** supramolecular three-dimensional net considering these interactions. (*c*) Resulting fourfold interpenetration of the individual **pcu** nets.

To gain a deeper insight on the potential assembly and organization of this molecule and study the interactions detected in the supramolecular network, a topological study [21,22] was performed with TOPOS [23–25] considering the molecules as nodes and the hydrogen bonds as linkers. Each molecule was found to be connected to six neighbours (one for each carboxylic acid, see electronic supplementary material, figure S3), giving rise to a uninodal three-dimensional net with **pcu** topology (**pcu **= primitive cubic, figure 3*b*). There are four independent three-dimensional supramolecular nets, which are interpenetrated (class Ia, *Z* = 4) as depicted in figure 3 and electronic supplementary material, figure S3. This dense three-dimensional web of interactions is responsible for the poorer solubility of compound **3** in its crystalline form and could affect the availability of this molecule when used as a drug if dispensed in its crystalline form.

**Figure 4.**
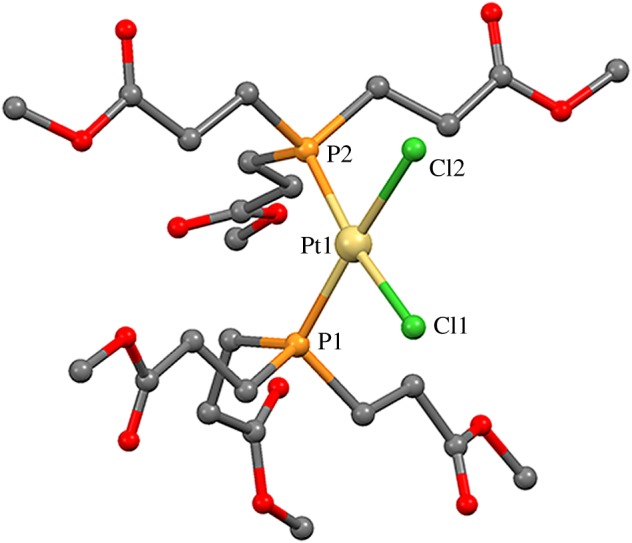
Molecular structure of complex **2.**

The crystal structure of complex **2** was also solved and corroborates all the other characterizations performed. Figure 4 shows the molecule in the asymmetric unit, with the Pt atom in a square planar geometry with an angle P1–Pt1–P2 of 104.98**°**, clearly more distorted from the ideal 90° than the equivalent angle in **1·1,25H_2_O.** The Pt–Cl and Pt–P distances are within the expected range for this kind of complex: (2.329–2.377 Å) [26,27] and (2.233–2.271 Å) [26,28]; and are very similar to the ones found in the reported structure for compound **1·1,25H_2_O** (table 2).

**Table 2.** Selected distances and angles for complexes **2** and **1·1,25H_2_O** [14].

	bond distances (Å)		bond angles (°)			
**2**	Pt1–P1	2.254(1)	P1–Pt1–P2	104.98(4)	P1–Pt1–Cl1	84.68(4)
	Pt1–P2	2.255(1)	P2–Pt1–Cl1	170.32(4)	P1–Pt1–Cl2	170.93(4)
	Pt1–Cl1	2.365(1)	P2–Pt1–Cl2	83.18(4)	Cl1–Pt1–Cl2	87.14(4)
	Pt1–Cl2	2.350(1)				
**1·1,25H_2_O**	Pt1–P1	2.2597(8)	P1–Pt1–P2	96.36(2)	P1–Pt1–Cl1	90.48(2)
	Pt1–P2	2.2455(8)	P2–Pt1–Cl1	173.15(2)	P1–Pt1–Cl2	174.62(2)
	Pt1–Cl1	2.3714(8)	P2–Pt1–Cl2	88.72(2)	Cl1–Pt1–Cl2	84.43(2)
	Pt1–Cl2	2.3588(8)				
	Pt2–P3	2.2539(7)	P3–Pt2–P4	95.36(2)	P3–Pt2–Cl3	89.84(2)
	Pt2–P4	2.2571(8)	P4–Pt2–Cl3	174.49(2)	P3–Pt2–Cl4	175.81(2)
	Pt2–Cl3	2.3616(9)	P4–Pt2–Cl4	88.80(2)	Cl3–Pt2–Cl4	85.99(2)
	Pt2–Cl4	2.3558(8)				

If we compare the supramolecular interactions found in the structures of compounds **1·1,25H_2_O** [14] and **2** with the ones found in **3**, we see that, in compound **2**, the packing is only achieved by much weaker hydrogen bonds. As expected, in compound **1·1,25H_2_O**, the supramolecular arrangement also entails hydrogen bonds comparable in strength to the ones found in **3**. However, in the crystal structure of **1·1,25H_2_O**, the disposition of the carboxylic groups in the molecule and the involvement of the interstitial water molecules in the supramolecular interactions prevent the formation of a network of hydrogen bonds similar to the one found in **3**, which results in a higher solubility in water.

### 2.3. Biomolecule interactions with the complexes

**Figure 5.**
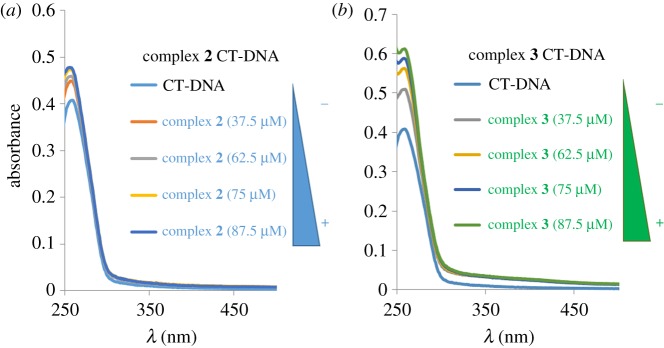
UV–visible analysis of CT-DNA interaction with increasing concentration of complex **2** (*a*) and complex **3** (*b*) in Tris–HCl buffer solution.

To estimate the nature of the supramolecular interaction of complexes **2** and **3** with calf thymus DNA (CT-DNA), we analysed the changes in the typical absorbance at 260 nm of CT-DNA using spectrophotometric titrations, varying the concentration of each complex (from 37.5 µM to 87.5 µM) with constant CT-DNA concentration (6.2 × 10^−5^ M) (figure 5).

Both complexes showed the same pattern of interaction. Increasing the complex concentration, the DNA absorption band at 260 nm increases, showing a known effect named hyperchromism. Cisplatin has been widely reported to produce a bathochromic shift from 260 to 265 nm [29] caused by perturbation resulting from covalent bonding between the Pt atom and the N7 atoms of the purine bases which is obviously not observed in complex **1** and **2**. Hyperchromism indicates a decrease in the degree of unwinding, whether produced by covalent binding or by non-covalent interaction [30].

**Figure 6.**
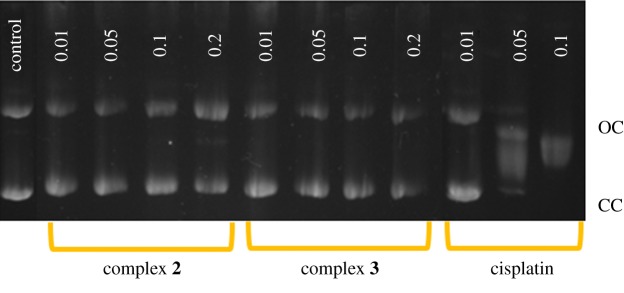
Electrophoresis in agarose gel of complex **2** (lines 2–5) and **3** (lines 6–9) with pBR322 at *r*_i_ = 0.001 to 0.2. Cisplatin (lines 10–12) and pBR322 control (line 1) have been included for comparative purposes.

To further support the different interaction of those complexes we also studied their interaction with the DNA plasmid supercoiled pBR322 (which contains two isoforms). Cisplatin has been reported to alter both forms of this plasmid to such a degree until they co-migrate at *r*_i_ = 0.02 (*r*_i_ = molar ratio Pt/nucleotide) in an electrophoresis experiment. The CC form (closed circular) is delayed because of the platination, and the OC form (open circular) slows down because of the unwinding. Complexes **2** and **3** (figure 6) do not produce any changes in the electrophoretic mobility of both forms, demonstrating that they do not have any binding affinity towards this DNA model.

Hydrodynamic methods such as determination of viscosity are sensitive to the change of length of DNA that allows one to shed some light on the nature of the interaction with DNA.

Complex **1** reactivity with a dsDNA has been recently studied in the presence of a model lipid membrane [31]. The results suggested that bonding of complex **1** to DNA leads to a much more compact structure than cisplatin's DNA bonding, revealing complex **1** interacts by hydrogen bonding on the hydrophilic regions. Based on this information and on the experiments carried out so far with these complexes, the determination of the viscosity produced by the complexes becomes an interesting tool to study the nature of their interaction with DNA. Specifically, the relationship between the relative viscosity and the contour length is of the form *L*/*L*_0 _= (*η*/*η*_0_)^1/3^, where *L*_0_ and *η*_0_ denote the apparent DNA molecular length and the solution viscosity, respectively, in the absence of a tested compound.

**Figure 7.**
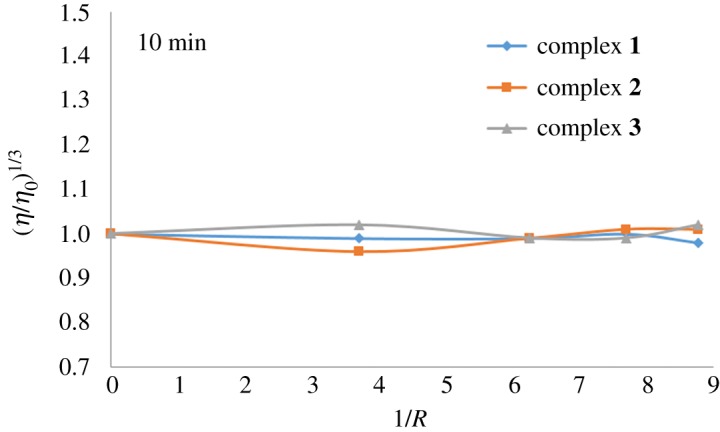
Effect of increasing amounts of compounds **1**, **2** and **3** on the viscosity of CT-DNA. The data were collected for [DNA] = 6.2 × 10^−5^ M at diverse 1/*R* ratios (*R* = [DNA]/[compound]).

A classical intercalator molecule like ethidium bromide causes a significant increase in the viscosity of the DNA solution due to the separation of base pairs at the interaction site and an increase in overall double helix length, whereas a groove binder agent like Hoechst 33258 does not appreciably lengthen the DNA helix and, therefore, causes less-pronounced changes or no changes at all in the viscosity of DNA solutions. A covalent binder case, for example cisplatin, causes a decrease in the relative viscosity of the DNA solution [32]. The values of cube root of the relative specific viscosity, (*η*/*η*_0_)^1/3^, versus the ratio of the complex concentration to DNA, 1/*R* (where *R* = [CT-DNA]/[compound]), in the absence and in the presence of compounds **1**, **2** and **3** are plotted in figure 7.

The three compounds exhibit no changes in the viscosity, indicative of non-covalent interaction (as the example described for Hoechst), which is also in agreement with the spectrophotometric studies performed with CT-DNA. Compound **2**'s structure does not show the same bonding site (the carboxyethyl group has been methylated), so the electrostatic interaction can be discarded and only hydrogen bonding can be possible, though much weakened.

The importance of TCEP as a reductor in biological systems, its significant role in the cisplatin crystallization with Atox1 and the fact that complex **3** reacts with glutathione in water to afford species prompted us to compare such interaction with protein models using both complexes **2** and **3**.

The selected models are: (i) egg white lysozyme (HEWL, hen egg white lysozyme), which is a protein broadly used as a model for interaction of metallodrugs [33] and (ii) bovine pancreatic RNase, often used as a model also for complex interaction with a ribonucleotide reductase enzyme [34]. In table 3, we have also compiled the *K*_HEWL_ and *K*_RNase_ data, calculated based on a pseudo first-order reaction of complexes **2** and **3** with the protein models. (All the data have been compiled and presented in detail in the Experimental section and electronic supplementary material, figures S4–S6.)

**Table 3.** Calculated values for: stability *K*, interaction with HEWL (*K*_HEWL_) and RNase (*K*_RNase_) of complexes **2, 3** and cisplatin.

complex	*K* (s^−1^)	*K*_HEWL_ (s^−1^)	*K*_RNase_ (s^−1^)
**2**	5.98 × 10^−5^	1.90 × 10^−4^	1.55 × 10^−4^
**3**	1.12 × 10^−4^	1.38 × 10^−4^	5.50 × 10^−4^
cisplatin	4.45 × 10^−5^ [35]	1.98 × 10^−4^	1.88 × 10^−4^

The constant values of interactions with both proteins for complexes **2** and **3** are very similar and also consistent with the stability values. In spite of the similarity, we can see that complex **3** (*trans* isomer) shows a slightly higher affinity for RNase.

### 2.4. Cytotoxicity

Cisplatin derivatives are widely used in the treatment of gastric cancer disease. To investigate the effect of these compounds *in vitro*, we studied the survival of the two most common human gastric adenocarcinoma cell lines (MKN45 and ST2957) after treatment with increased doses of each compound and compared with cisplatin. We analysed cell survival 72 h after treatment with cisplatin (0–150 µM), compound **2** (0–150 µM) or compound **3** (0–100 µM). Our results indicated that as we have previously described, both cell lines MKN45 and ST2957 showed cisplatin sensitivity with different IC_50_: 7 versus 20 µM, respectively [36].

**Figure 8.**
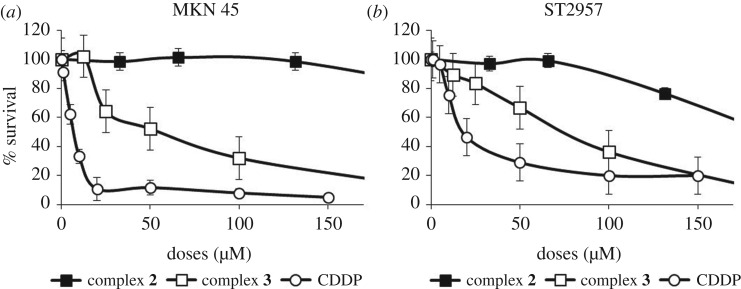
Survival of MKN45 and ST2957 cells after treatment with CDDP or new complexes **2** and **3**. (*a*) MKN45 and (*b*) ST2957 cell lines were treated with increasing amounts of CDDP (open circles), complex **2** (filled squares, μM) or complex **3** (open squares). Viability was quantified using the crystal violet method 72 h after treatment. The percentage is shown as relative to the number of cells without treatment. Data represent the means of two experiments performed in quadruplicate.

Compound **2** has no effect on viability in either MKN45 or ST2957 cell lines. By contrast, compound **3** decreased survival on both cell lines in a dose-dependent manner reaching IC_50_ of 50 µM for MKN45 cells versus 73 µM for ST2957 cells. These results suggest that compound **3** has a biological effect on gastric cancer cells (figure 8). Table 4 shows the IC_50_ required for each compound. Our data indicate that complex **3** needs a much higher dose than cisplatin to kill tumour cells. However, the dose used is in the same range as that of carboplatin used to kill these cells. Furthermore, carboplatin is an approved clinical drug in use, so our results could represent a step forwards in new drug design.

**Table 4.** IC_50_ values (μM) for complexes **2**, **3** and cisplatin.

cell line	cisplatin (µM)	complex 2 (µM)	complex 3 (µM)
MKN45	7	132	50
ST2957	20	>150	73

According to the values of table 4, we think that the viability data cannot be only produced by a DNA lesion, but also other molecular targets might be involved. These results are of high importance to overcome the problems with cisplatin treatment and its secondary side effects, and have opened up a new field of investigation that must extend the knowledge of its mechanism of action not only through apoptosis but also through new cell routes described for these particular cancer cells [37].

## 3. Experimental

### 3.1. Material and methods

Monodimensional ^195^Pt-NMR, ^13^C-NMR and ^1^H-NMR experiments were performed in DMSO-d_6_ and D_2_O using a Bruker AMX-300 (300 MHz) spectrometer at room temperature (25°C). Elemental analyses were performed with a PerkinElmer 2400 Series II microanalyzer. IR was performed with a PerkinElmer Model 283 spectrophotometer with an ATR accessory (Miracle Single Reflection Horizontal). UV–visible spectroscopy was performed with a Thermo Fisher Scientific Evolution 260 Bio spectrophotometer. Mass spectrometry was performed with a QSTAR de ABSciex by electrospray.

### 3.2. Single-crystal X-ray diffraction

X-ray diffraction data collection was done at 296(2) K for compounds **2** and **3** with a Bruker Kappa Apex II diffractometer using graphite-monochromated Mo-K*α* radiation (*λ *= 0.71073 Å). Details on the data collection and refinement are gathered in electronic supplementary material, tables S2 and S3. CCDC 1519230 and 1519231 contain the supplementary crystallographic data for compounds **2** and **3**. The data can be obtained free of charge from the Cambridge Crystallographic Data Centre via www.ccdc.cam.ac.uk/structures.

### 3.3. Cytotoxicity assay

Viability was determined using a crystal violet-based staining method, as described previously [38]. Briefly, 5 × 10^4^ cells per well were seeded in 24 multiwell dishes, treated with cisplatin (0–150 µM) complex **2** (0–150 µM) or complex **3** (0–100 µM) for 72 h and fixed with 1% glutaraldehyde. After they were washed in 1× phosphate-buffered saline, the remaining cells were stained with 0.1% crystal violet. Colorimetric assay using 595 nm Elisa was used to estimate the number of cells per well. Complexes **2** and **3** were dissolved in water. IC_50_ values were calculated by using the GraphPad Prism program. We used nonlinear regression to fit the data to the log (inhibitor) versus response (variable slope) curve.

### 3.4. Synthetic procedures

#### 3.4.1. Synthesis of *cis*-[Pt(TCEP)_2_Cl_2_], complex **1**

A 4 ml aliquot of a degassed water solution containing 37.2 mg of K_2_PtCl_4_ was added to 50.2 mg of TCEP previously dissolved in 2 ml of H_2_O. The addition was performed under an argon atmosphere and the reaction was stirred at room temperature for 24 h. The final white solid was filtered off and washed with cold water, 766.36 g mol^−1^. Yield: 73.9%. Calculated analysis for C_18_H_30_Cl_2_O_12_P_2_Pt: 28.21% C; 3.95% H; 0% H; Found: 27.81% C; 3.92% H; 0% N. ^1^H-NMR (DMSO, 300 MHz) *δ* 2.30 (H1, 12 H), *δ* 2.55 (H2, 12 H). ^13^C-NMR (DMSO, 300 MHz) *δ* 19.0 (C1), *δ* 28.7 (C2), *δ* 172.7 (C3). ^31^P-NMR (DMSO, 300 MHz) *δ* 4.18 (s, ^1^*J*_PPt _= 3482.9). ^195^Pt-NMR (DMSO, 300 MHz) *δ* −4420.5 (t, ^1^*J*_PtP _= 3486.36) MHz) *δ* 19.0 (C1), *δ* 28.7 (C2), *δ* 172.7 (C3). ^31^P-NMR (DMSO, 300 MHz) *δ* 4.18 (s, ^1^*J*_PPt _= 3482.9). ^195^Pt-NMR (DMSO, 300 MHz) *δ* −4420.5 (t, ^1^*J*_PtP _= 3486.36). Anal. (%) calcd. for C_18_H_30_Cl_2_O_12_P_2_Pt. C: 28.21, H: 3.95, N: 0. Found. C: 27.81, H: 3.92, N: 0. ESI-MS [M–Cl]^+^: *m/z* 731.

#### 3.4.2. Synthesis of *cis*-[Pt(tmTCEP)_2_Cl_2_], complex **2**

TCEP (49.8 mg) previously dissolved in 2 ml of MeOH was added to a suspension of 36.5 mg of K_2_PtCl_4_ in methanol under an argon atmosphere. The mixture was stirred at room temperature for 2 days. The final white solid was filtered and washed with water and methanol. 850.52 g mol^−1^. Yield: 73.9%. ^1^H-NMR (CD_3_OD, 300 MHz) *δ* 2.44 (H1, dt, *J *= 7.2 *y* 3.2 Hz, 12 H), *δ* 2.76 (H2, dt, *J = *7.2 *y* 1.8 Hz, 12 H), *δ* 3.30 (H4, s, 18 H); ^13^C-NMR (CD_3_OD, 300 MHz) *δ* 20.81 (C1). *δ* 29.90 (C2). *δ* 52.68 (C4). *δ* 174.0 (C3); ^31^P-NMR (CD_3_OD, 300 MHz) *δ* 4.87 ppm (s, ^1^*J*_PPt _= 3525.24). ^195^Pt-NMR (CD_3_OD, 300 MHz) *δ* −4433.08 ppm (t, ^1^*J*_PtP _= 3522.15). Anal. (%) calcd. for C_24_H_42_Cl_2_O_12_P_2_Pt. C: 33.89, H: 4.98, N: 0. Found. C: 33.83, H: 4.82, N: 0. ESI-MS [M–Cl]^+^: *m/z* 815.14.

#### 3.4.3. Synthesis of *trans*-[Pt(TCEP)_2_Cl_2_], complex **3**

*Cis*-[Pt(TCEP)_2_Cl_2_], **2** (20 mg) was heated in 5 ml of D_2_O, at 100°C for 20 min. Lowering the temperature of the final solution to room temperature gave the full precipitation of a yellow solid. The solid was isolated by filtration and washed with cold water and dried in a drying oven. 766.36 g mol^−1^. Yield: 92%. ^1^H-NMR (D_2_O, 300 MHz) *δ* 2.35 (H1, 12 H), *δ* 2.83 (H2, 12 H) 12.3 ppm (O–H, broad, 6H and interchange with D_2_O); ^13^C-NMR (D_2_O, 300 MHz) *δ* 16.2 (C1), *δ* 28.5 (C2), *δ* 172.7 (C3); ^31^P-NMR (D_2_O, 300 MHz) *δ* 9.25; ^195^Pt-NMR (D_2_O, 300 MHz) *δ* −4357.51. Anal. (%) calcd. for C_18_H_30_Cl_2_O_12_P_2_Pt. C: 28.21, H: 3.95, N: 0. Found. C: 28.63, H: 3.78, N: 0. ESI-MS [M+Na]^+^ : *m/z* 788.99.

### 3.5. Sample preparation for interaction with model proteins

To evaluate the biological behaviour of complexes **2** and **3**, they were initially dissolved in DMSO in a 5 mM concentration. For all experiments, the desired concentration of complexes was achieved by dilution of the stock DMSO solution with aqueous buffer. Cisplatin stock solutions were prepared in 5 mM NaClO_4_ and diluted in Milli-Q water. All the solutions and buffers were previously tempered to 37°C. Afterwards, the freshly prepared complex solutions were mixed in a thermoshaker at 37°C with Milli-Q water (for stability K) [16] and aqueous buffer Tris (for DNA/model protein solutions). The studies never exceeded 0.5% DMSO (v/v) in the final solution. Control experiments with DMSO were performed and no changes in the spectra of the model proteins or CT-DNA were observed.

### 3.6. Complexes and CT-DNA interaction

Binding experiments of complexes **2** and **3** to CT-DNA were analysed twice by spectrophotometric titrations, keeping constant the CT-DNA concentration (6.2 × 10^−5^ M) while varying the concentration of each complex (0–87.5 µM) and monitoring the changes in the typical absorbance of CT-DNA at 260 nm after equilibration (10 min, at 37°C).

### 3.7. Viscosity studies

The viscosity experiments were carried out in automated AND viscometer model SV-1A, at constant temperature of 25.0 ± 0.1°C using a water bath with thermostat control. The concentration of CT-DNA was maintained constant (6.2 × 10^−5^ M), with increasing concentrations of complexes **1**, **2** and **3** (0–87.5 µM) to ratios in the range of 0–8.8 (1/*R* = [compound]/[CT-DNA]). Complexes **2** and **3** were initially dissolved in DMSO : water at a 5 mM concentration. The dilution was performed using Tris–HCl buffer to a final volume of 2 ml containing a maximum amount of 1.0% of DMSO.

Viscosity experiments were recorded twice, taking two different measurements for each experiment (time 0 and 10 min).

### 3.8. pBR322 interaction with the complexes

The DNA-binding studies were performed in a total volume of 20 µl. The DNA aliquots containing 8 ml of DNA-pBR322 (10 ng ml^−1^ stock) in 10 mM Tris–HCl (pH 7.6) and 1 mM EDTA were incubated with the platinum compounds at several *r*_i_ values (0.01–0.5) using the corresponding amount of platinum from either a 5 or 50 µM stock solution. (Stock solutions were prepared in water and used fresh.) The samples were incubated at 37°C for 24 h, after which time 2 µl of a loading dye containing 50% glycerol, 0.25% bromophenol blue and 0.25% xylene cyanol was added. The whole of the sample (20 µl) was loaded in the wells of a 0.8% agarose gel. Electrophoresis was carried out for a period of 2.5 h at approximately 50 V. After electrophoresis, the gel was immersed in 800 ml of Millipore water containing 64 µl of a 10 mg ml^−1^ stock solution of ethidium bromide for 30 min to stain the DNA.

### 3.9. UV–visible kinetics experiments with protein

To investigate the interaction of cisplatin and complexes **2** and **3** with plasma proteins, electronic spectra of the protein models HEWL and RNase A at 10^−5^ M were recorded before and after the addition of complexes **2** and **3** for 24 h and at 37°C, and at a stoichiometric ratio of 10 : 1 and 3 : 1 (metal to protein) with the same results. The experimental time-dependent spectral profile was analysed as a pseudo first-order reaction by plotting the variation of the absorbance as a function of time (absorption profiles in electronic supplementary material, figures S4–S6, and plots in electronic supplementary material, figure S6).

## 4. Conclusion

We report the synthesis of three platinum complexes using TCEP and tmTCEP (where esterification and coordination take place in the same step). The crystal structures demonstrated *cis* configuration for complex **2** and *trans* for complex **3**. A comparative study of the supramolecular interactions in the solid state has been performed. The *cis* complex **1** affords slow isomerization to complex **3** in water solution, while complexes **2** and **3** showed good stability in water solution and Tris–HCl buffer. The reactivity of these two stable compounds is higher when they interact with model proteins than with DNA. The complexes do not react when they face the secondary DNA structure model (pBR322), and with CT-DNA they act very differently compared with cisplatin.

## Supplementary Material

Additional structural and reactivity data for complexes 2 and 3

## Data accessibility

The crystallographic data can be obtained free of charge from the Cambridge Crystallographic Data Centre via www.ccdc.cam.ac.uk/structures; CCDC 1519230 and 1519231 contain the supplementary crystallographic data for compounds **2** and **3**. Data for the paper and cif data are in the electronic supplementary material.
