# Supplementary material for: Using phosphine ligands with a biological role to modulate reactivity in novel platinum complexes
Source: R Soc Open Sci. 2018 Feb 21;5(2):171340. doi: 10.1098/rsos.171340 (PMC5830740; doi:10.1098/rsos.171340)
Supplement: Additional structural and reactivity data for complexes 2 and 3 [file rsos171340supp1.docx]

Received 00th January 20xx,

1. Inorganic Chemistry department. Universidad Autónoma de Madrid.
2. SIDI. Universidad Autonoma de Madrid
3. Instituto de Investigaciones biomédicas Albert Sols.

Electronic Supplementary Information (ESI) available: [details of any supplementary information available should be included here]. See DOI: 10.1039/x0xx00000x

Accepted 00th January 20xx

DOI: 10.1039/x0xx00000x

www.rsc.org/

Using phosphine ligands with a biological role to modulate its reactivity in novel platinum complexes

Marcelo Echeverri,^a^ Amparo Alvarez-Valdés,^a^ Francisco Navas,^a^ Josefina Perles^b^ Isabel Sanchez-Perez, ^c^ and A.G. Quiroga^a^

.

Contenido

[Figure SM1. Plots of the absorbance variation as a function of time for the solutions of the complexes 2 and 3. 2](#_Toc501640184)

[Figure SM2. Hydrogen bonds in 3 yielding the supramolecular net with pcu topology. Depicted in cyan, the interactions between one of the molecules and its six neighbours. In red, the remaining hydrogen bonds from each of these adjacent molecules to their remaining five neighbours. 2](#_Toc501640185)

[Figure SM3. Schematic view of the positions related to the unit cell of the four interpenetrated supramolecular pcu nets (in blue, orange, magenta and green). 3](#_Toc501640186)

[Table SM1: Supramolecular interactions found in compounds 2 and 3. The strongest hydrogen bonds in 3 (yielding the supramolecular 3D net) are highlighted in bold letters. 3](#_Toc501640187)

[Table SM2: Sample data, crystal data, refinement and data collection for complex 2. 4](#_Toc501640188)

[Table SM3: Sample data, crystal data, collection and structure refinement data for complex 3. 5](#_Toc501640189)

[Figure SM4. Time dependence UV-Vis analysis of Lysozyme interaction with complex 2 and 3 in Tris-HCl buffer solution from t=0 to 24h. 6](#_Toc501640190)

[Figure SM5. Time dependence UV-Vis analysis of RNase interaction with complex 2 and 3 in Tris-HCl buffer solution from t=0 to 24h. 7](#_Toc501640191)

[Figure SM6. Time dependence UV-Vis analysis of Lysozyme and RNase interaction with cisplatin in Tris-HCl buffer solution from t=0 to 24h. 7](#_Toc501640192)

[Figure SM7. Plots of the variation of the absorbance as a function of time of complex 2, 3 and cisplatin with lysozyme and RNase. 8](#_Toc501640193)


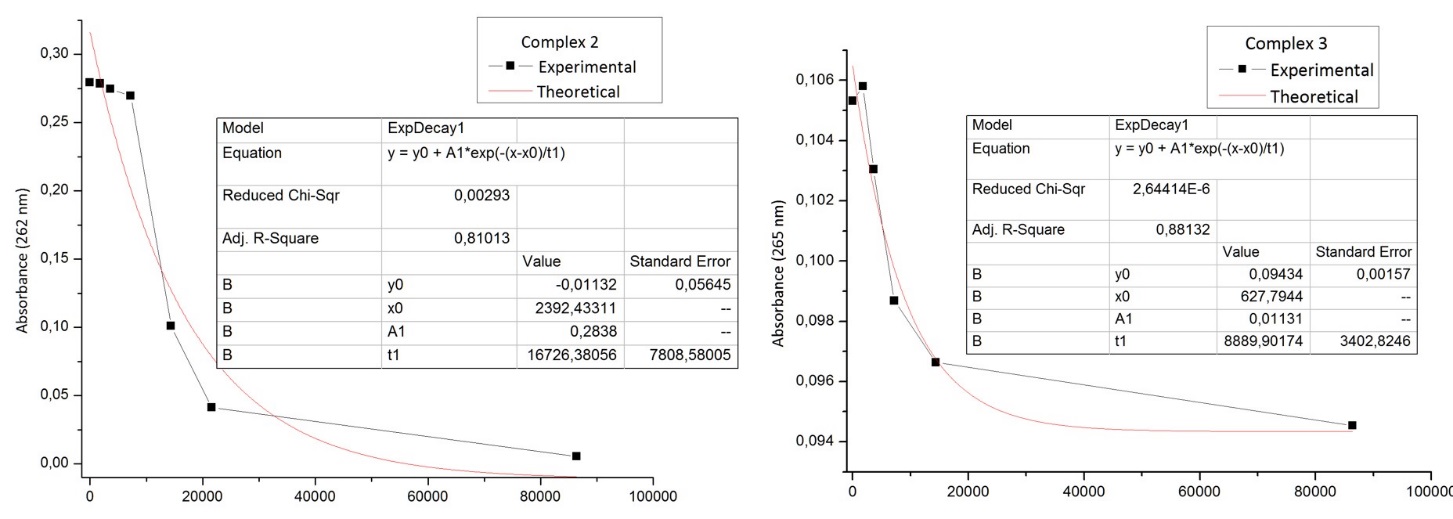


# Figure SM1. Plots of the absorbance variation as a function of time for the solutions of the complexes 2 and 3.


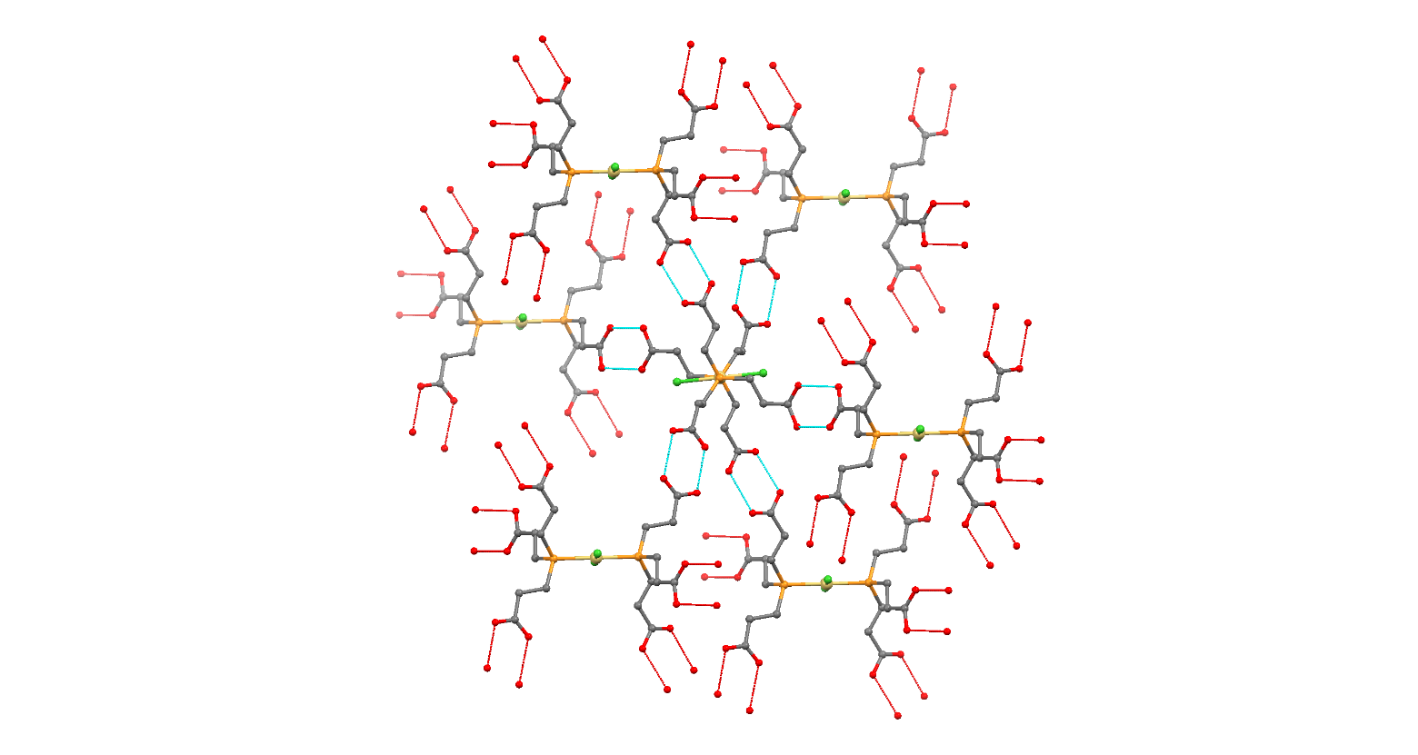


# Figure SM2. Hydrogen bonds in 3 yielding the supramolecular net with pcu topology. Depicted in cyan, the interactions between one of the molecules and its six neighbours. In red, the remaining hydrogen bonds from each of these adjacent molecules to their remaining five neighbours.


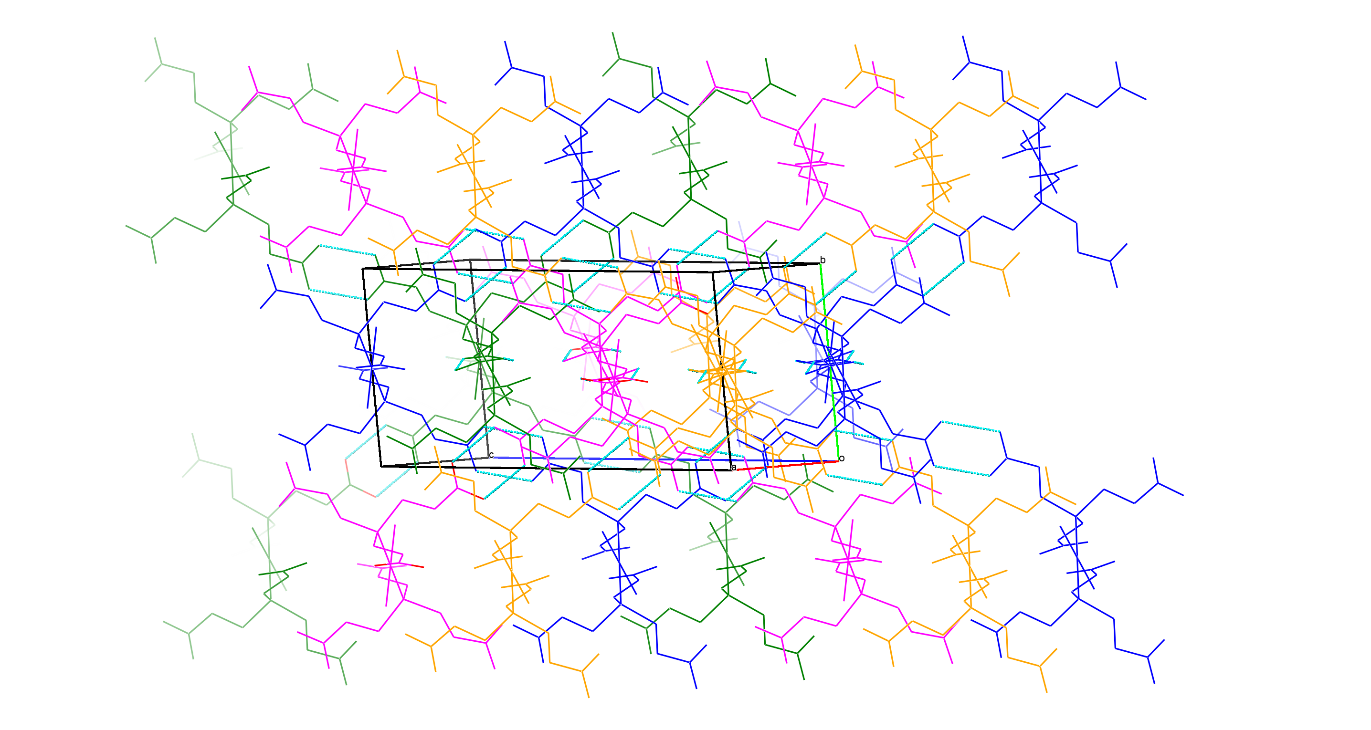


# Figure SM3. Schematic view of the positions related to the unit cell of the four interpenetrated supramolecular pcu nets (in blue, orange, magenta and green).

# Table SM1: Supramolecular interactions found in compounds 2 and 3. The strongest hydrogen bonds in 3 (yielding the supramolecular 3D net) are highlighted in bold letters.

| **2** | | | | |
| --- | --- | --- | --- | --- |
|  | **Donor-H (Å)** | **Acceptor-H (Å)** | **Donor-Acceptor (Å)** | **Angle (°)** |
| C1-H1B^...^Cl1 | 0.97 | 2.88 | 3.396(4) | 114.4 |
| C5-H5A^...^Cl1 | 0.97 | 2.78 | 3.712(4) | 161.8 |
| C9-H9A^...^Cl1 | 0.97 | 2.79 | 3.349(4) | 117.4 |
| C10-H10A^...^O4 | 0.97 | 2.57 | 3.377(7) | 141.2 |
| C10-H10B^...^O10 | 0.97 | 2.37 | 3.278(5) | 156.6 |
| C12-H12B^...^O12 | 0.96 | 2.58 | 3.514(8) | 164.1 |
| C12-H12C^...^O2 | 0.96 | 2.61 | 3.075(8) | 110.3 |
| C13-H13A^...^Cl2 | 0.97 | 2.97 | 3.458(5) | 112.2 |
| C16-H16C^...^O12 | 0.96 | 2.65 | 3.40(1) | 135.0 |
| C17-H17B^...^O8 | 0.97 | 2.62 | 3.443(5) | 143.4 |
| C20-H20A^...^O5 | 0.96 | 2.60 | 3.476(9) | 152.2 |
| C21-H21A^...^O8 | 0.97 | 2.63 | 3.441(6) | 141.1 |
| C22-H22A^...^Cl2 | 0.97 | 2.80 | 3.462(5) | 126.0 |
| C24-H24A^...^O5 | 0.96 | 2.56 | 3.451(8) | 155.3 |
| C24-H24B^...^O9 | 0.96 | 2.62 | 3.567(8) | 171.1 |
|  | | | | |
| **3** | | | | |
| **O2-H1O^...^O11** | **0.82** | **2.19** | **2.715(6)** | **121.6** |
| **O4-H4O^...^O9** | **0.82** | **1.85** | **2.667(6)** | **172.2** |
| **O6-H6O^...^O7** | **0.82** | **1.89** | **2.663(5)** | **157.3** |
| **O8-H8O^...^O5** | **0.82** | **1.84** | **2.638(6)** | **163.1** |
| **O10-H10O^...^O3** | **0.82** | **1.82** | **2.632(6)** | **170.3** |
| **O12-H11O^...^O1** | **0.82** | **1.82** | **2.602(6)** | **157.6** |
| C1-H1A^...^Cl1 | 0.97 | 2.79 | 3.411(6) | 122.4 |
| C1-H1B^...^O8 | 0.97 | 2.61 | 3.562(7) | 167.8 |
| C8-H8B^...^Cl1 | 0.97 | 2.79 | 3.482(6) | 129.2 |
| C10-H10A^...^O11 | 0.97 | 2.56 | 3.408(7) | 146.0 |
| C11-H11A^...^Cl2 | 0.97 | 2.89 | 3.570(6) | 127.8 |
| C14-H14A^...^O6 | 0.97 | 2.47 | 3.316(7) | 145.4 |
| C14-H14B^...^Cl2 | 0.97 | 2.89 | 3.559(6) | 127.1 |
| C16-H16B^...^O7 | 0.97 | 2.42 | 3.325(7) | 154.4 |
| C17-H17A^...^Cl2 | 0.97 | 2.91 | 3.610(6) | 130.0 |

# Table SM2: Sample data, crystal data, refinement and data collection for complex 2.

| **Chemical formula** | C_24_H_42_Cl_2_O_12_P_2_Pt | |
| --- | --- | --- |
| **Formula weight** | 850.51 | |
| **Temperature** | 296(2) K | |
| **Wavelength** | 0.71073 Å | |
| **Crystal size** | 0.08 x 0.11 x 0.21 mm | |
| **Crystal habit** | clear colourless prismatic | |
| **Crystal system** | Triclinic | |
| **Space group** | *P* -1 | |
| **Unit cell dimensions** | *a* = 7.4312(2) Å | α = 77.439(1)° |
|  | *b* = 14.4597(5) Å | β = 85.159(1)° |
|  | *c* = 16.0531(5) Å | γ = 84.854(1)° |
| **Volume** | 1673.14(9) Å^3^ |  |
| **Z** | 2 | |
| **Density (calculated)** | 1.688 Mg/cm^3^ | |
| **Absorption coefficient** | 4.503 mm^-1^ | |
| **F(000)** | 848 | |
| **Theta range for data collection** | 1.45 to 25.35° | |
| **Index ranges** | -8<=h<=8, -17<=k<=17, -19<=l<=19 | |
| **Reflections collected** | 55421 | |
| **Independent reflections** | 6089 [R(int) = 0.0390] | |
| **Coverage of independent reflections** | 99.6% | |
| **Absorption correction** | multi-scan | |
| **Max. and min. transmission** | 0.7146 and 0.4515 | |
| **Structure solution technique** | direct methods | |
| **Structure solution program** | SHELXS-97 (Sheldrick, 2008) | |
| **Refinement method** | Full-matrix least-squares on F^2^ | |
| **Refinement program** | SHELXL-97 (Sheldrick, 2008) | |
| **Function minimized** | Σ w(F_o_^2^ - F_c_^2^)^2^ | |
| **Data / restraints / parameters** | 6089 / 0 / 376 | |
| **Goodness-of-fit on F^2^** | 1.016 | |
| **Final R indices** | 5432 data; I>2σ(I) | R1 = 0.0219,  wR2 = 0.0571 |
|  | all data | R1 = 0.0297,  wR2 = 0.0724 |
| **Weighting scheme** | w=1/[σ^2^(F_o_^2^)+(0.0502P)^2^+0.6421P] where P=(F_o_^2^+2F_c_^2^)/3 | |
| **Largest diff. peak and hole** | 0.718 and -0.901 eÅ^-3^ | |
| **R.M.S. deviation from mean** | 0.215 eÅ^-3^ | |

# Table SM3: Sample data, crystal data, collection and structure refinement data for complex 3.

| **Chemical formula** | C_18_H_30_Cl_2_O_12_P_2_Pt | | |
| --- | --- | --- | --- |
| **Formula weight** | 766.35 | | |
| **Temperature** | 296(2) K | | |
| **Wavelength** | 0.71073 Å | | |
| **Crystal size** | 0.04 x 0.10 x 0.14 mm | | |
| **Crystal habit** | clear colourless prismatic | | |
| **Crystal system** | Triclinic | | |
| **Space group** | *P* -1 | | |
| **Unit cell dimensions** | *a* = 7.7735(2) Å | | α = 88.782(1)° |
|  | *b* = 8.9759(2) Å | | β = 86.022(1)° |
|  | *c* = 19.0294(4) Å | | γ = 84.508(1)° |
| **Volume** | 1318.34(5) Å^3^ | |  |
| **Z** | 2 | | |
| **Density (calculated)** | 1.931 Mg/cm^3^ | | |
| **Absorption coefficient** | 5.703 mm^-1^ | | |
| **F(000)** | 752 | | |
| **Theta range for data collection** | 2.51 to 25.35° | | |
| **Index ranges** | -9<=h<=9, -10<=k<=10, -22<=l<=22 | | |
| **Reflections collected** | 48941 | | |
| **Independent reflections** | 4820 [R(int) = 0.0599] | | |
| **Coverage of independent reflections** | 99.9% | | |
| **Absorption correction** | multi-scan | | |
| **Max. and min. transmission** | 0.8040 and 0.5023 | | |
| **Structure solution technique** | direct methods | | |
| **Structure solution program** | SHELXS-97 (Sheldrick, 2008) | | |
| **Refinement method** | Full-matrix least-squares on F^2^ | | |
| **Refinement program** | SHELXL-97 (Sheldrick, 2008) | | |
| **Function minimized** | Σ w(F_o_^2^ - F_c_^2^)^2^ | | |
| **Data / restraints / parameters** | 4820 / 0 / 325 | | |
| **Goodness-of-fit on F^2^** | 1.045 | | |
| **Final R indices** | 3267 data; I>2σ(I) | R1 = 0.0243,  wR2 = 0.0525 | |
|  | all data | R1 = 0.0491,  wR2 = 0.0606 | |
| **Weighting scheme** | w=1/[σ^2^(F_o_^2^)+(0.0221P)^2^+2.0456P] where P=(F_o_^2^+2F_c_^2^)/3 | | |
| **Largest diff. peak and hole** | 0.767 and -0.496 eÅ^-3^ | | |
| **R.M.S. deviation from mean** | 0.106 eÅ^-3^ | | |


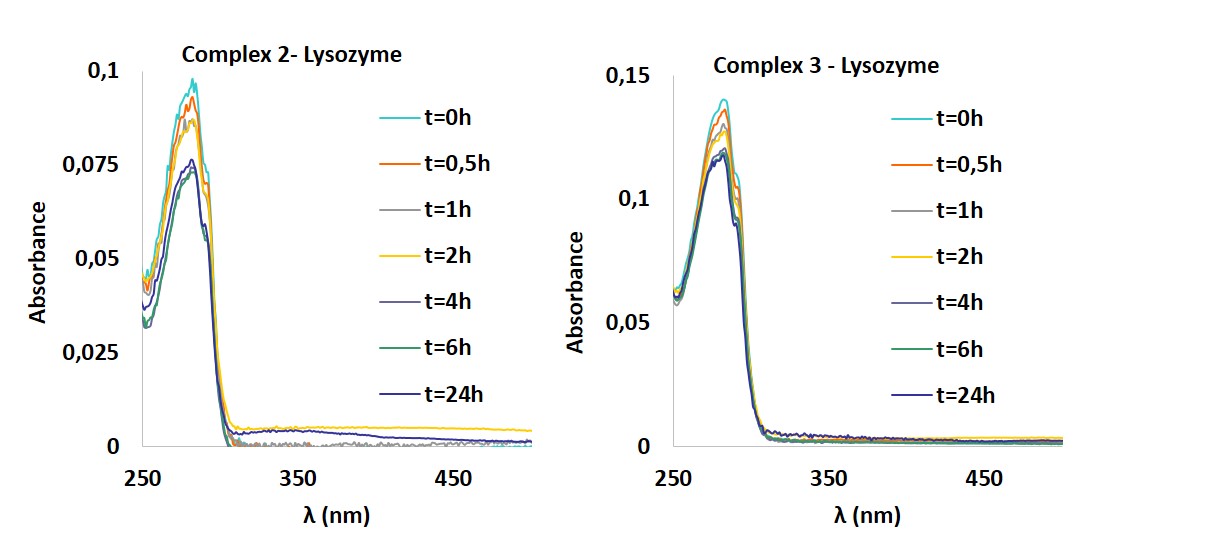


# Figure SM4. Time dependence UV-Vis analysis of Lysozyme interaction with complex 2 and 3 in Tris-HCl buffer solution from t=0 to 24h.


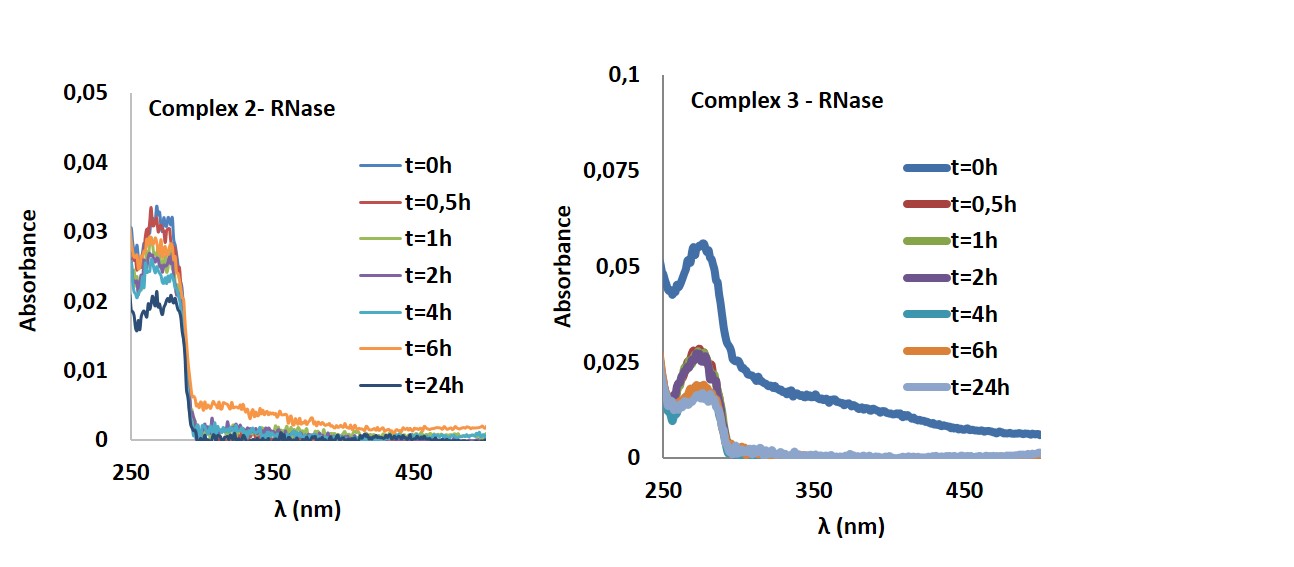


# Figure SM5. Time dependence UV-Vis analysis of RNase interaction with complex 2 and 3 in Tris-HCl buffer solution from t=0 to 24h.


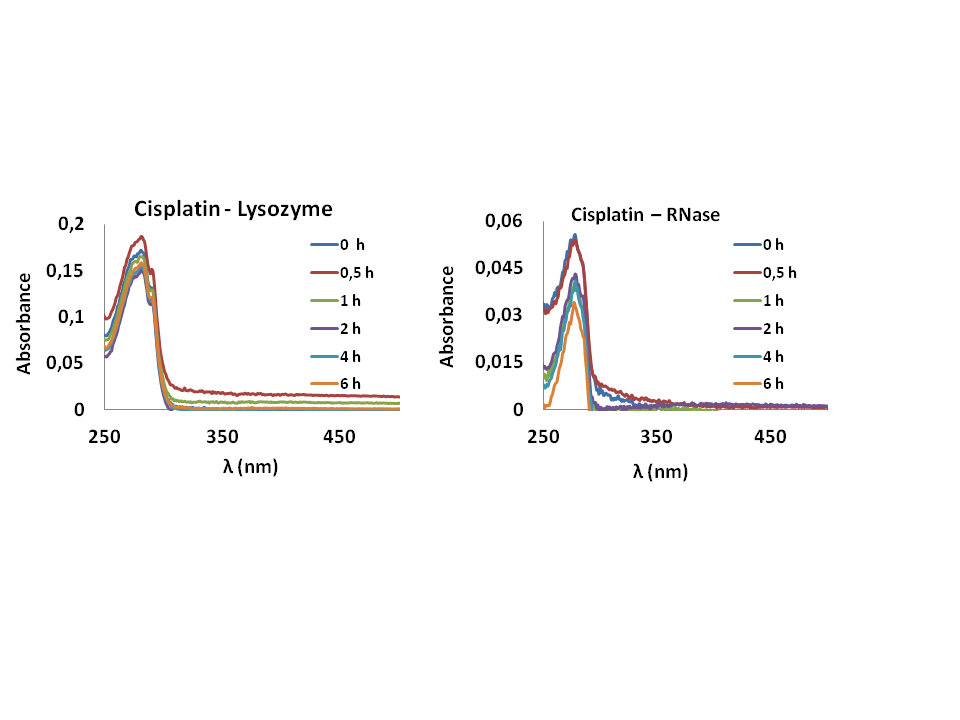


# Figure SM6. Time dependence UV-Vis analysis of Lysozyme and RNase interaction with cisplatin in Tris-HCl buffer solution from t=0 to 24h.


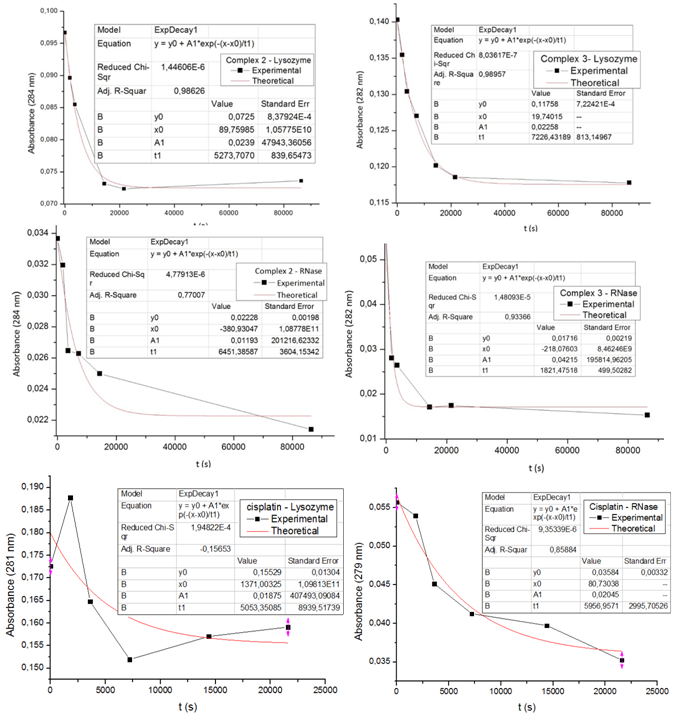


# Figure SM7. Plots of the variation of the absorbance as a function of time of complex 2, 3 and cisplatin with lysozyme and RNase.
